# SARS-CoV-2 Infections in Animals: Reservoirs for Reverse Zoonosis and Models for Study

**DOI:** 10.3390/v13030494

**Published:** 2021-03-17

**Authors:** Tessa Prince, Shirley L. Smith, Alan D. Radford, Tom Solomon, Grant L. Hughes, Edward I. Patterson

**Affiliations:** 1 NIHR Health Protection Unit in Emerging and Zoonotic Infections, Department of Clinical Infection, Microbiology and Immunology, University of Liverpool, Liverpool L69 7TX, UK; tsolomon@liverpool.ac.uk (T.S.); Grant.Hughes@lstmed.ac.uk (G.L.H.); 2 Institute of Infection, Veterinary and Ecological Sciences, University of Liverpool, Liverpool L69 7BE, UK; shirley@liverpool.ac.uk (S.L.S.); Alanrad@liverpool.ac.uk (A.D.R.); 3 Walton Centre NHS Foundation Trust, Liverpool L9 7LJ, UK; 4 Centre for Neglected Tropical Disease, Departments of Vector Biology and Tropical Disease Biology, Liverpool School of Tropical Medicine, Pembroke Place, Liverpool L3 5QA, UK; 5 Department of Biological Sciences, Brock University, St. Catharines, ON L2S 3A1, Canada

**Keywords:** COVID-19, SARS-CoV-2, animals, reverse zoonosis, intermediate host

## Abstract

The recent SARS-CoV-2 pandemic has brought many questions over the origin of the virus, the threat it poses to animals both in the wild and captivity, and the risks of a permanent viral reservoir developing in animals. Animal experiments have shown that a variety of animals can become infected with the virus. While coronaviruses have been known to infect animals for decades, the true intermediate host of the virus has not been identified, with no cases of SARS-CoV-2 in wild animals. The screening of wild, farmed, and domesticated animals is necessary to help us understand the virus and its origins and prevent future outbreaks of both COVID-19 and other diseases. There is intriguing evidence that farmed mink infections (acquired from humans) have led to infection of other farm workers in turn, with a recent outbreak of a mink variant in humans in Denmark. A thorough examination of the current knowledge and evidence of the ability of SARS-CoV-2 to infect different animal species is therefore vital to evaluate the threat of animal to human transmission and reverse zoonosis.

## 1. Introduction

The final month of 2019 presented the world with a new respiratory illness, the first reports of which emerged from the city of Wuhan, China. We now know that the disease was COVID-19, caused by a novel coronavirus (2019-nCoV) [1], afterward renamed severe acute respiratory syndrome coronavirus 2 (SARS-CoV-2) [2]. It has subsequently spread globally and caused more than a million deaths.

There have been five human coronavirus epidemics in the last twenty years: HCoV-NL63 and HCoV-HKU1, which cause mild respiratory symptoms and circulated through the population, and the more serious SARS (2003), Middle East respiratory syndrome (MERS) (2012), and, now, SARS-CoV-2 in 2019. SARS-CoV originated in bats and was transmitted to humans via civet cats [3] while MERS originated in camels [4]. While it is currently unknown the precise route by which SARS-CoV-2 is transmitted from animals to humans, it is argued to have a bat origin [1,5]. Early speculation suggested two routes by which the SARS-CoV-2 originated: either natural selection in a human following zoonotic acquisition or natural selection in an intermediate animal host prior to zoonotic transmission to a human [6]. Many of the first cases of SARS-CoV-2 visited a live animal market in Wuhan, which houses many live animals, suggesting that an animal at the market may have transmitted the virus to these first individuals [1]; however, it is now believed this was not the location of the first zoonotic transmission and rather acted as a super spreader event.

**Figure 1:**
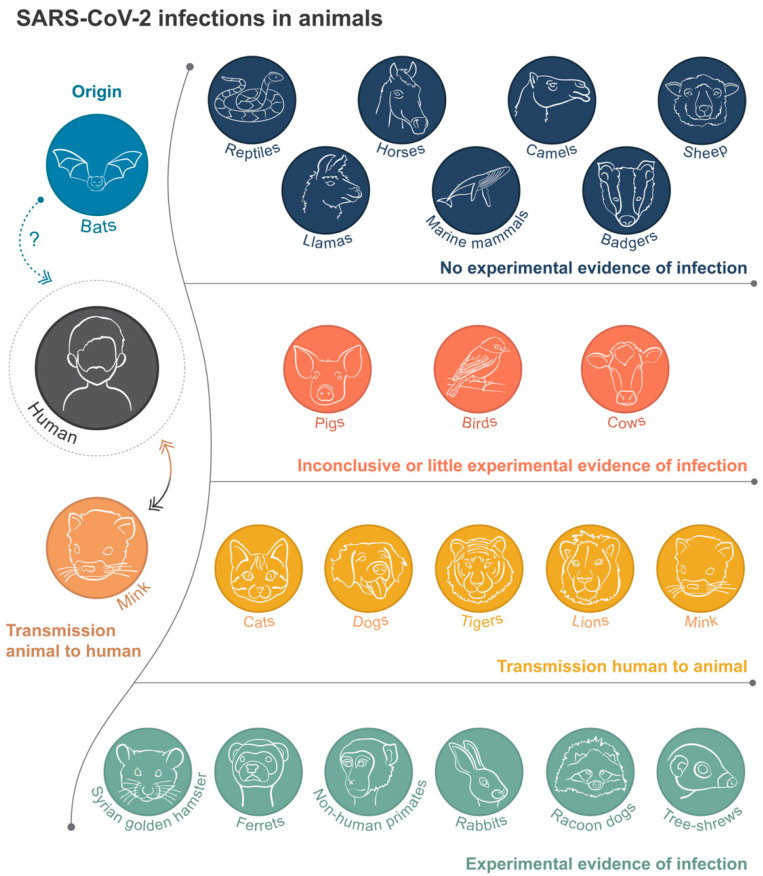
Diagram representing the current evidence on the infection and transmission of SARS-CoV-2 in animals and the relationship to human infections. Prepared by Manuela Bernardi.

Coronaviruses, such as the one that caused COVID-19, are enveloped, single-stranded, positive-sense RNA viruses. They possess the largest genome of the RNA viruses with four open reading frames (ORFs) that code for the main structural proteins: the Spike (S), Membrane (M), Envelope (E), and Nucleocapsid (N) [7]. Like its 2003 predecessor, SARS-CoV-2 is now known to adhere to the host angiotensin-converting enzyme-2 (ACE2) receptor via its receptor-binding domain (RBD) of the viral S protein [8]. ACE-2 receptors can be found on a variety of cell types in the human body—in particular, the oral and nasopharyngeal mucosae, lung, gastrointestinal tract, liver, kidney, and brain [9]. Modeling studies have predicted that SARS-CoV-2 can bind to several animal species’ ACE2 receptors, including those of pigs, cats, ferrets, and nonhuman primates, with varying accuracy [10]. However, these modeling studies do not replace the experimental evidence of SARS-CoV-2 infections in these animals, which we focus upon here in an attempt to understand which animals are susceptible to SARS-CoV-2 infection (Figure 1).

## 2. SARS-CoV-2 Infections in Animals

### 2.1. In the Wild

The SARS-CoV-2 virus sequence is 96% identical to the bat coronavirus RaTG13, detected in *Rhinopholus affinis* in Yunnan Province in China [1]. Similarly, another bat coronavirus, RmYN02, has shown 97.2% similarity in the longest gene-encoding region, 1ab [11]. This has led experts to believe that, at some point, SARS-CoV-2 likely emerged as a result of a recombination event between a bat coronavirus and an unknown coronavirus, possibly in an unknown intermediate host [12]. Early in the pandemic, there was speculation that pangolins (*Manis javanica*) could have been such an intermediate host [13]. SARS-CoV-2-like coronaviruses were present in Malayan pangolins seized in antismuggling operations in China [14] and single-cell sequencing identified pangolin cell types likely to be permissive to the virus [15]. In contrast, a Malaysian study failed to identify any coronaviruses in pangolins rescued from the wild or confiscated from smugglers before they entered the illegal trade network. This suggests that coronaviruses found in Malayan pangolins in China may have been acquired through contact with humans and other animals in the illegal trade network. However, the lack of infection in the latter case does not prove that they are not involved in some way [16]. To date, there have been no identified cases of SARS-CoV-2 in wild animals of any kind.

### 2.2. Livestock Farming

Although there is no evidence of infection with SARS-CoV-2 in animals in the wild, there is concern about the potential for a reverse zoonotic event occurring from humans to animals living in close proximity to humans and the establishment of a permanent viral reservoir in animals. Livestock farming is an area of particular concern, given the close human–animal contact, particularly in some regions of the world, and the potential for the high stocking density of some farmed animal species and the threat to food supply chains. Livestock such as cattle, sheep, and other ruminants can be infected by a variety of coronaviruses [7]; however, the evidence concerning the SARS-CoV-2 infection of cattle is currently limited to a single study [17]. Ulrich et al. experimentally infected six cattle (*Bos taurus*) via the intranasal route with a 1 × 10^5^ tissue culture infectious dose 50% (TCID_50_) and monitored the animals for twenty days. After 24 h, three naïve contact animals were introduced to examine transmission. Low-level viral shedding was observed in two of the six experimentally infected cattle, but no transmission was seen in in-contact animals [17]. Despite their apparent vulnerability to infection, to date, there is no evidence of a human naturally transmitting the virus to cattle or other livestock. Likewise, there is currently no evidence yet that sheep, horses, donkeys, camels, or llamas [18] can become infected with SARS-CoV-2. Further research to explore the susceptibility of cattle and other livestock animals is needed to determine if they can act as reservoirs of the virus.

The greatest evidence to date for reverse zoonosis is in minks (*Neovison vison* and *Mustela vison*), farmed for their fur. There have been several reports of virus outbreaks on mink farms in Spain, Denmark, the USA [19,20], the Netherlands [19], France, Italy, Sweden, Canada, Greece, Lithuania [20], and Poland [21]. First reported in the Netherlands, the virus was introduced by infected farm workers [22]. Minks on infected farms were culled, and weekly testing of all animals that died was introduced, revealing that the virus has since evolved due to the widespread transmission between minks [23]. In a total of 16 mink farms that were affected, 68% of employees had evidence of SARS-CoV-2 infection. An in-depth investigation of the Dutch outbreaks suggested that at least two farm workers acquired the virus in turn directly from the animals [22,23,24]. This is the only evidence presently that suggests a possible transmission from an animal directly to humans since the beginning of the pandemic. However, an analysis of the genetic sequences of SARS-CoV-2 in patients living near the mink farms indicated they were not related to the clusters seen at the mink farms [23].

In an analysis of viral genomes isolated from minks, three recurrent mutations in the spike receptor-binding domain have been identified: Y453F, F486L, and N501T. These mutations are found at a low frequency in viruses circulating in humans, suggesting that they could be responsible for the adaptation of the virus to infect minks [25].

In November 2020, reports emerged of a mink “variant” of SARS-CoV-2 in five mink farms in North Jutland in Denmark. The mink variant appears to have infected 12 humans (named “cluster 5”); eight cases had a direct link to mink farms, while four cases were from the community. “Cluster 5” represents a variant of the virus with a combination of mutations not previously seen. Human cases infected with the mink variant presented with similar clinical presentation and transmission properties [26,27]. All the viruses isolated from cases in “cluster 5” possessed the Y453F mutation. To date, 214 human cases of COVID-19 have been identified in Denmark that have been associated with the mink variant of SARS-CoV-2. The Statens Serum Institute in Denmark performed microneutralisation antibody assays and found an average 3.53-fold reduction in neutralising antibody titres to the mink variant compared to the wild-type virus. This result suggests that there could be an impact on antibody-mediated immunity acquired through infection or vaccination [27] and has led to a call for the widespread culling of all minks in Denmark [28]. Danish public health authorities have recommended the enhanced sequencing of human and mink infections and heightened the surveillance of the human population in North Jutland, alongside movement restrictions for the population living there. However, since then, scientists have stated that the mutations themselves are not a cause for concern; they do not imply that the virus will transmit more efficiently between people. Meanwhile, other human variants (e.g., the “British” and “South African” variants) have become more concerning in that regard [29]. Furthermore, no further cases of the “cluster 5” variant have been identified in people since September [30]. Further studies are required to determine the impact this could have on the efficacy of vaccine candidates. Taken together, this evidence demonstrates that determining if other mustelid species such as badgers, weasels, polecats, martens, and otters could act as an intermediate host of the virus is required [31].

Given the concern over livestock farming, rabbits, which are also farmed for their meat and fur, have also been investigated. Rabbits (*Oryctolagus cuniculus*) have been inoculated with SARS-CoV-2 and observed for 21 days. None of the rabbits exhibited any clinical signs, but the animals shed infectious virus from their nose and throat and were shown to seroconvert. This was performed using young New Zealand white rabbits and thus may not reflect infections in different ages or breeds; therefore, further investigation into their potential as viral hosts is appropriate [32].

### 2.3. Zoos

One of the early surprises during the pandemic was the apparent infection of a tiger in the Bronx Zoo in New York [33,34]. Subsequently, another four tigers and three lions at the same zoo were found to have SARS-CoV-2 [35]. The infectious virus was isolated from both the tracheal wash fluid and feces of both animals [36]. Interestingly, whole-genome sequencing revealed that both cat species were infected by different SARS-CoV-2 strains, suggesting they acquired the virus through two different transmission events. The testing of zoo keepers that were in contact with the animals revealed genetic and epidemiological similarities between the keeper and animal virus isolates, indicating human-to-tiger transmission [35]. However, a clear route of transmission was not identified for the infection in the lions. As a result, the zoo implemented social distancing measures and the use of masks/visors to protect the animals from acquiring the infection from their carers or members of the public [35]. A lion in Spain also tested positive for SARS-CoV-2 RNA [20]. Other big cat species can be infected; a puma in a zoo in South Africa tested positive for viral RNA after contact with an infected handler [36], and one snow leopard tested positive for viral RNA in a zoo in Kentucky, USA [20]. Furthermore, reports have also emerged of the infection of three gorillas in a zoo in San Diego, CA, USA [37], demonstrating that extended safety measures should be taken in zoos to prevent the infection spreading from humans to known susceptible animals.

### 2.4. Companion Animals

#### 2.4.1. Cats

Much like their larger counterparts, domestic cats are also susceptible to SARS-CoV-2 infection. Cats have their own feline coronaviruses, FCoV I and II [7], which are only distantly related to SARS-CoV-2. In addition, SARS-CoV RNA was detected in cats from a live animal market in Guangzhou in 2004 [38]. Experimentally, cats have been infected with SARS-CoV-2 and shown to transmit the virus to one another through respiratory droplets [39,40,41]. Since the pandemic began, there have been sporadic cases of SARS-CoV-2 infections in domestic cats around the world. For example, SARS-CoV-2 RNA has been found in 47.1% of 17 tested cats in the USA [42,43], in cats in Belgium, Greece, Switzerland, Brazil [20], Italy [44], Hong Kong [45], France [46], Canada [20], Germany, Spain [47,48], Japan [20], Russia [20], the Netherlands [22], and in the UK [49]. A survey in Chile found that cats appeared to excrete viral RNA for a shorter duration than humans. In addition, the similarity of the genetic sequences of viral RNA detected in humans and their pet cats indicated direct human-to-animal transmission, though whether animal-to-animal transmission occurred was not determined [50].

In addition to the presence of viral RNA, evidence of prior infections (i.e., neutralizing antibodies) were demonstrated in cats. A study in Wuhan where cats were sampled both during and after the outbreak revealed 11 out of 141 cats had neutralizing antibodies to the virus using plaque reduction neutralization tests (PRNTs), while using an in-house ELISA found 15 cats were positive for the antibody. No serological reactivity was found between SARS-CoV-2 and FCoV I and II [51]. Another group evaluated 66 pet cats and 21 stray cats across China using a commercial ELISA and found that no cats from Wuhan had antibodies, contradicting the Wuhan observations [52]. This led to the further examination of 630 feline serum samples collected before November 2019 and 423 samples collected after the pandemic began across 20 cities in China. These were evaluated using the same commercial ELISA. All cat samples taken both before and after the outbreak were seronegative [53].

In France, the status of 22 cats from the homes of people suspected or confirmed to have had COVID-19 revealed one cat was positive for viral RNA and, subsequently, seroconverted (measured using an adapted commercial ELISA) [46]. An additional small study evaluated nine cats in a veterinary community of 20 students in which two students tested positive for SARS-CoV-2 RNA and eleven others had suspected COVID-19 symptoms. However, using an immunoprecipitation assay, they found that no cats had SARS-CoV-2-specific antibodies one month after the first index case was reported, and no viral RNA was detected in the cats [54]. In contrast, a further seroprevalence study demonstrated that 23.5% (8/34) of cats from COVID-19-positive households had antibodies, while one out of 16 cats from households of unknown status were positive for the antibodies [55].

Moreover, during the Dutch mink farm outbreaks, stray cats around the farm were tested for the virus, with one out of 24 cats testing positive for viral RNA and seven out of 24 cats testing positive for antibodies against the virus using an in-house microneutralization assay [22]. Whether they acquired the virus through contact with infected humans, minks, or infectious fomites is not known. The role of cats in the transmission of SARS-CoV-2 in between mink farms has yet to be evaluated [31].

A large Italian seroprevalence study provided further supporting evidence of SARS-CoV-2 infection in cats. Neutralizing antibodies, with titres varying from 1:20 to 1:160, were found in 3.9% of (6/152) tested cats in Italy [56]. No juvenile cats in this study were found to have evidence of past infections, implying that infection may be linked to age in this population. The study also found no connection between cat seropositivity and the COVID-19 household infection status. This study was performed using plaque reduction neutralisation tests (PRNTs), currently the gold standard for the detection of neutralizing antibodies.

Most recently, some evidence has emerged to suggest that cats previously infected with SARS-CoV-2 develop a partial nonsterilizing immunity and, as such, can be reinfected by the virus. However, on secondary infection, cats do not appear to shed the virus in sufficient quantities to transmit the virus onwards to naïve cats [57].

Notably, each study varied with respect to the sampling strategy of animals from both confirmed and suspected households, numbers of animals tested, and the choice of test used to detect antibodies, perhaps explaining the disparities in the results. While currently, there is no evidence to suggest cats can transmit the infection to humans, this trend in domestic cats suggests routine testing and vigilance regarding pet cats would be a sensible precaution, and well-designed studies are required to reveal the true prevalence of disease in cats worldwide.

#### 2.4.2. Dogs and Other Pets

Like cats, dogs have their own distantly related coronaviruses, including canine enteric coronavirus and canine respiratory coronavirus [7]. Experimentally infected dogs shed little to no virus, indicating they would be unlikely to contract the virus and transmit it [39]. However, seven dogs in the USA have now tested positive for SARS-CoV-2 RNA, while eight have tested positive for antibodies to the virus [43]. In Hong Kong, two out of 15 dogs from COVID-19-positive households tested positive for the virus and seroconverted [58]. Both dogs were asymptomatic throughout the course of the infection. Interestingly, one of the households had another dog that remained negative for the virus throughout, suggesting transmission between dogs is not high [58]. Viral RNA was also found in two dogs in Japan [20], and one dog tested positive for the virus in the Netherlands [59]. A further two dogs tested positive for SARS-CoV-2 in Germany [20], one dog in Canada [20], and in Argentina, and Mexican reports have emerged of dogs positive for SARS-CoV-2 RNA [20].

In France, a serological survey of companion animals showed 15.4% of dogs (2/13) from COVID-19-positive households had neutralizing antibodies to SARS-CoV-2. No dogs from households with an unknown COVID-19 status had neutralizing antibodies. To combat the impact of variable antibody responses and differences in their detection, this study utilized multiple methods to identify neutralizing antibodies [55]. In contrast, in a veterinary campus, 12 dogs had no antibodies of the virus, despite close contact with humans with SARS-CoV-2 [54]. Supporting evidence for infections in dogs in Italy showed that 3.4% (13/388) of the tested dogs had neutralizing antibodies to the virus, but none were positive for viral RNA [56]. Dogs with neutralizing antibodies were significantly more likely to have come from a COVID-19-positive household. Interestingly, a higher proportion of male dogs were seropositive for antibodies compared to female canines, inferring there may be a behavioural or hormonal aspect to the acquisition of the virus in dogs. Sadly, none of the current studies reported any information regarding the neutered status of the animals, limiting our understanding of how gender and hormones may impact the SARS-CoV-2 infection in domestic pets.

In Spain, the evidence is further contrasting, with little support for the virus found in dogs [47]. However, 40 dogs with pulmonary signs were evaluated alongside 20 healthy dogs from COVID-19-positive households for evidence of SARS-CoV-2 infection. No dogs were found to have SARS-CoV-2 RNA, but five healthy and one sick dog had antibodies to SARS-CoV-2 [60]. A further survey in China evaluated 90 beagles, 250 street dogs, and 147 pet dogs and found none had any antibodies to SARS-CoV-2 [52].

Taken together, this information indicates that further research is required to assess the extent and situations that might lead dogs to contract the virus from their human contacts and if they transmit it to other animals. Furthermore, working dogs such as bomb-sniffing dogs or COVID-19-sniffing dogs could be adversely affected if they can acquire the virus. Unlike some human infections, there is currently no indication that infection leads to hyposmia/anosmia in dogs [61]. There are currently no reports that dogs can transmit the virus to humans. However, as with cats, these studies used a variety of sampling methods, animal numbers, and testing strategies, making comparisons difficult. Multiple studies noted the increased risk of exposure to dogs in COVID-19-positive households. While there is no evidence of transmission back to humans or among pets, it would be prudent to include pets in self-isolation measures. As with mink farms, any risk of zoonotic infection would likely be highest where large groups of cats or dogs are housed together, such as in kennels, catteries, and rescue shelters.

One guinea pig and two rabbits from three COVID-19-positive households in Spain were tested for viral RNA, and none of these were positive for the virus [47].

## 3. Further Experimental Proof of Permissiveness to Infection

With many species’ ACE2 receptors predicted to recognize SARS-CoV-2 [10], much research has been invested in identifying which animals are permissive to infection; in particular rodents, pigs, ducks, chickens, rabbits, racoon dogs, and tree shrews. While mice were not predicted to have ACE2 receptors that could bind SARS-CoV-2 [10], researchers experimentally infected deer mice (*Peromyscus maniculatus*) and found viral replication occurred but the infection was not lethal, and mice did not develop clinical signs. However, the transmission between mice through direct contact was observed, indicating it may be a useful model to study the viral pathogenesis and transmission. While it is theoretically possible that these mice could act as an intermediate reservoir, presently, there is no indication of deer mouse infections in nature [62,63].

Single-cell screening for SARS-CoV-2 target cells (i.e., those that have the ACE2 receptor) in a variety of animals has shown multiple susceptible cell types in the domestic pig (*Sus scrofa domesticus*) [15]. Pigs inoculated with SARS-CoV-2 and their contacts tested negative for the virus by PCR and did not develop antibodies, signifying they are not susceptible to infection [39]. Likewise, pigs could not be infected with SARS-CoV-2 intranasally [64]. This work contrasts with a study showing the live virus isolated from oronasally infected pigs and the development of a humoral immune response [65]. Others have shown that pig airway epithelial cells do not support the viral replication [18]. These conflicting studies suggest further research into the ability of pigs to become infected with and transmit the virus is needed. No data on the naturally acquired infection in pigs has come to light as yet.

While other respiratory viruses are known to be carried by chickens and waterfowl, little is known of whether SARS-CoV-2 can be carried by birds. SARS-CoV-2 replicates poorly in ducks and chickens and does not result in the generation of neutralizing antibodies [39]. This result has been replicated in chickens by others [64], and the same was seen for ducks, turkeys, quails, and geese for both SARS-CoV-2 and MERS-CoV [66]. Taken together, it seems unlikely that poultry can act as an intermediate reservoir of the virus.

Racoon dogs (*Nyctereutes procyonoides*), which are most closely related to foxes, are also susceptible to infection with SARS-CoV-2, with six out of nine experimentally infected racoon dogs able to produce the infectious virus. Furthermore, transmission of the virus from infected dogs to two-thirds of the contact animals was demonstrated. Some authors have suggested that, as part of the fur trade in China, this animal could have played a role in the early stages of the pandemic [67]. Given the data supporting infection with SARS-CoV-2 in their canid cousin, the domestic dog, further investigation of the *caninae*, including racoon dogs, wolves, and foxes, would be reasonable.

The tree shrew (*Tupaia belangeris*) has been evaluated as a COVID-19 model. This animal is genetically similar to primates and has been used as a model for other viral infections. Tree shews show little clinical signs of infection, but some histopathology and low-level viral replication occurs [68]. However, compared to other models such as mice, the tree shrew demonstrated lesser susceptibility to infection and would not be an optimal experimental model. However, this is does not mean that it could not act as an intermediate host of the virus [68].

White-tailed deer (*Odocoileus virginianus*) have been intranasally inoculated with the virus and shown to efficiently transmit the virus to naïve deer. The deer had subclinical infections and shed the virus through their nasal secretions and their feces. Of note, the virus used to infect the deer was an isolate from a Malayan tiger infected with the virus [35,69]. Further research into the susceptibility of related species should therefore be conducted.

## 4. Experimental Models of COVID-19 Disease

As the pandemic progresses and many countries are in their “second wave”, if not “third wave” of the virus, the hunt for an appropriate animal model to study the COVID-19 pathology and identify therapeutics and vaccine candidates has intensified. Several animals have already been assessed for their use as disease models.

While rodents have been shown in many cases to become infected with other coronaviruses [70], there is no suggestion yet of SARS-CoV-2 infection in wild rodents. Several studies have examined their permissiveness to infection in vivo as an experimental model of COVID-19, with the majority requiring genetic modification in order to express human ACE2 proteins or viral adaptation to recognize murine ACE2. The predominantly used mouse model is a transgenic model adapted to express human ACE-2 (K18-hACE-2) [71]. This model was recently used to demonstrate that infection with influenza followed by a coinfection with SARS-CoV-2 results in a more severe disease [72]. A further transgenic mouse model used experimentally is the HFH4-hACE-2 mouse [73]. This model has shown similar pathology to that seen in human COVID-19, and the surviving mice demonstrated a resistance to reinfection, with a neutralizing antibody response. Others have infected BALB/c aged mice with a mouse-adapted strain of the virus (SARS-CoV-2-MA10) [74]. Another rodent, the golden Syrian hamster (*Mesocricetus auratus*), exhibits signs of disease and can transmit the virus to other hamsters through close contact [75]. Similarly, Chinese hamsters (*Cricetulus griseus*) have also been developed as a COVID-19 model [76].

An alternative experimental model of SARS-CoV-2 infection that has been assessed for use is the ferret (*Mustela putorius furo*) [39,77,78]. Ferrets exhibit signs of the disease, including a loss of appetite and fever [39]. In addition, ferrets can also transmit the virus (seen both using environmental isolates from Wuhan and human isolates) through both direct contact and transmission through respiratory droplets over more than one meter distance [79]. The virus is found in the saliva, nasal washes, urine, and stool up to eight days after infection [39,77,78]. In contrast to these experimental infections, ferrets that were in prolonged close contact with humans with confirmed or probable SARS-CoV-2 infection did not acquire the virus from their human handlers. It is likely that both the virus and host genetic mutations pose a significant barrier to the transmission of the virus from humans to ferrets, suggesting that ferrets do not pose a significant threat for transmission of the virus in nature [80]. These findings contrast with the observations seen between farmed minks and their human handlers, where the proof of transmission between humans and minks is clear [22]. Furthermore there is a report of a pet ferret acquiring the infection in Slovenia, most likely from its infected human owner [20].

The close genetic relationship between humans and other nonhuman primates (NHPs) such as macaques, grivets, and marmosets means they have been employed to model the COVID-19 disease and, in particular, the immune response to the virus. Repeated infections in rhesus macaques (*Macaca mulatta*) have shown that viral loads from infected monkeys reduced during the secondary infection implying some immunity following the initial infection [81,82]. Viral shedding has been examined in different aged groups of cynomolgus macaques (*Macaca fascicularis*), with the shedding duration extended in older macaques compared to younger ones [83]. In African green monkeys (*Chlorocebus sabeaeus*), a much lower dose of virus can be used to establish the disease than that required for other primate models [84,85]. Finally, new world monkeys such as marmosets (*Callithrix jacchus*) have been compared to old world monkey models and found that the marmoset was the least susceptible to SARS-CoV-2 infection, while the rhesus macaques and cynomolgus macaques were the most susceptible [86].

## 5. Areas for Further Investigation

SARS-CoV-2, shed from humans, can be found in wastewater [87]. What threat this may pose to aquatic life and those animals residing close to contaminated water has yet to be determined. Using an ACE2 receptor modeling approach, marine mammal susceptibility to infection has been evaluated, and fifteen species of seals, whales, and dolphins were identified as potentially vulnerable [88]. There is currently no data to support the presence of the virus in mammals exposed to wastewater contamination. This suggests that some surveillance of species in areas where the wastewater treatment is suboptimal may be important in the future. However, other enveloped RNA viruses have been shown to experience declines in viral titres upon exposure to seawater, implying that the salt content and natural dilution that would occur in seawater would likely minimize the risk of the spread of SARS-CoV-2 to aquatic life [89].

Invertebrate species such as mosquitos have long been known to be important vectors of existing and emerging diseases, but no evidence yet exists this the case for coronaviruses. SARS-CoV-2 does not replicate in *Aedes* mosquito cells in vitro or in the live *Aedes* mosquitos captured in Wuhan during the pandemic [90], while both *Aedes* and *Culex* mosquitos injected with the virus do not support viral replication [91]. Biting midges (*Culicoides sonorensis*) and two *Culex* species (*Culex tarsalis* and *quinquefasciatus*) when allowed to feed on infected blood do not support the replication of SARS-CoV-2. Likewise, the cell lines established from these species do not support viral replication [92]. Taken together, the data suggests that biting insects ectoparasites do not carry and transmit the virus.

Finally, while not a direct infection of the animal, the role of animals and their byproducts (e.g., milk, meat, fur, and feathers) as inanimate carriers of the virus (fomites) has begun to be explored, with the carriage in fish held at 4 °C demonstrated [93], ice cream in China testing positive for the virus [94], and carriage for up to three weeks in refrigerated and frozen meat demonstrated experimentally [95]. The threat of viral emergence from live animal markets has long been highlighted; however, the present pandemic has generated questions over the Western industrial approach to the meat/fur trade and the role it plays in the transmission of viruses between people. With many reports of outbreaks of the infection in meat-processing factory workers [96], the implementation of health and safety policies will be important in limiting the spread of the infection. Meat-processing plants are ideal environments for spread of viruses; low temperatures, close contact between humans, the presence of feathers, feces, and dirt, combined with water, provide ideal conditions for the generation of infectious aerosols. While it is unlikely that food is a major source of infection, it appears there is the potential for fresh/frozen contaminated food to enter the supply chain, as has been observed with other viruses [97].

## 6. Conclusions

Since the SARS-CoV-2 outbreak has spread globally, the threat of a reverse zoonosis from humans to animals has become a distinct possibility. Currently, the best proof of transmission from humans to animals has been in farmed minks and domestic cats and dogs. The infection of domestic pets appears to be limited, with no evidence to suggest they can transmit the virus back to humans or to other species. However, there is some indication to suggest that minks can transmit the virus back to humans, posing a risk of continued reinfection from the virus circulating on mink farms and indicating that investigations into other mustelid species is important. The increased vigilance and screening of such farmed mammals could prevent the circulation of the virus between humans and animals and eliminate all other potential sources of the virus. However, it should be noted that many of the studies discussed here are yet to be peer reviewed, and many suffer from small population sizes, differing methods used, and sampling strategies. In the absence of greater understanding, it would seem wise to be particularly vigilant where large groups of susceptible species are housed, such as farms, kennels, and catteries.

Understanding where the SARS-CoV-2 adapted to humans is critical for our understanding and prevention of future outbreaks of not only COVID-19 but, also, other potential diseases. The best suggestion is that the virus evolved from a bat coronavirus; however, no intermediate host has yet been identified. The role of live animal markets in the emergence of new zoonotic diseases has long been recognized. It is unlikely that the COVID-19 pandemic will encourage changes in the culture around meat trading in known hot spots, as it would require changes in the political, economic, and cultural landscapes of regions already under considerable economic pressures. The continued human destruction of rural habitats and climate change will likely exacerbate the frequency of spillover events of emerging viruses from animals to humans. In the meantime, the continued surveillance of such wet markets and wildlife living at urban–rural borders is necessary to help understand the zoonotic transmission of viruses and prevent further emerging epidemics.

It is possible that SARS-CoV-2 may never be entirely eradicated, partly due to incomplete global vaccination and varying degrees of immunity such vaccines may provide. As yet, we do not know how long any immunity, whether vaccine-induced or due to prior infection, will last. Global coordination in vaccine rollouts and the development of variant-resistant vaccines are likely necessary to control the caseloads [98], in addition to the continued surveillance of SARS-CoV-2 in animals. With the exception of minks, it is encouraging that domestic animals such as cats and dogs are unlikely to act as a reservoir for continued infection in humans upon a background of rolling vaccinations. In addition to vaccinations, the ongoing surveillance of farmed, domestic, and wild animals must continue, alongside the interrogation of SARS-CoV-2 sequencing data in both humans and animals, in order to bring the pandemic under control and restore life to a modicum of normality.
